# First person – Ekaterina Gribkova

**DOI:** 10.1242/bio.062379

**Published:** 2025-12-05

## Abstract

First Person is a series of interviews with the first authors of a selection of papers published in Biology Open, helping researchers promote themselves alongside their papers. Ekaterina Gribkova is first author on ‘ Octopus ‘hypnosis’: inducing tonic immobility for studying local sensorimotor responses and arm-sucker coordination’, published in BiO. Ekaterina is a postdoctoral research associate in the lab of Dr Rhanor Gillette at the University of Illinois at Urbana-Champaign, Urbana, USA, investigating neuroethology and computational modelling, with a particular focus on bridging the gap between biological and artificial intelligence.


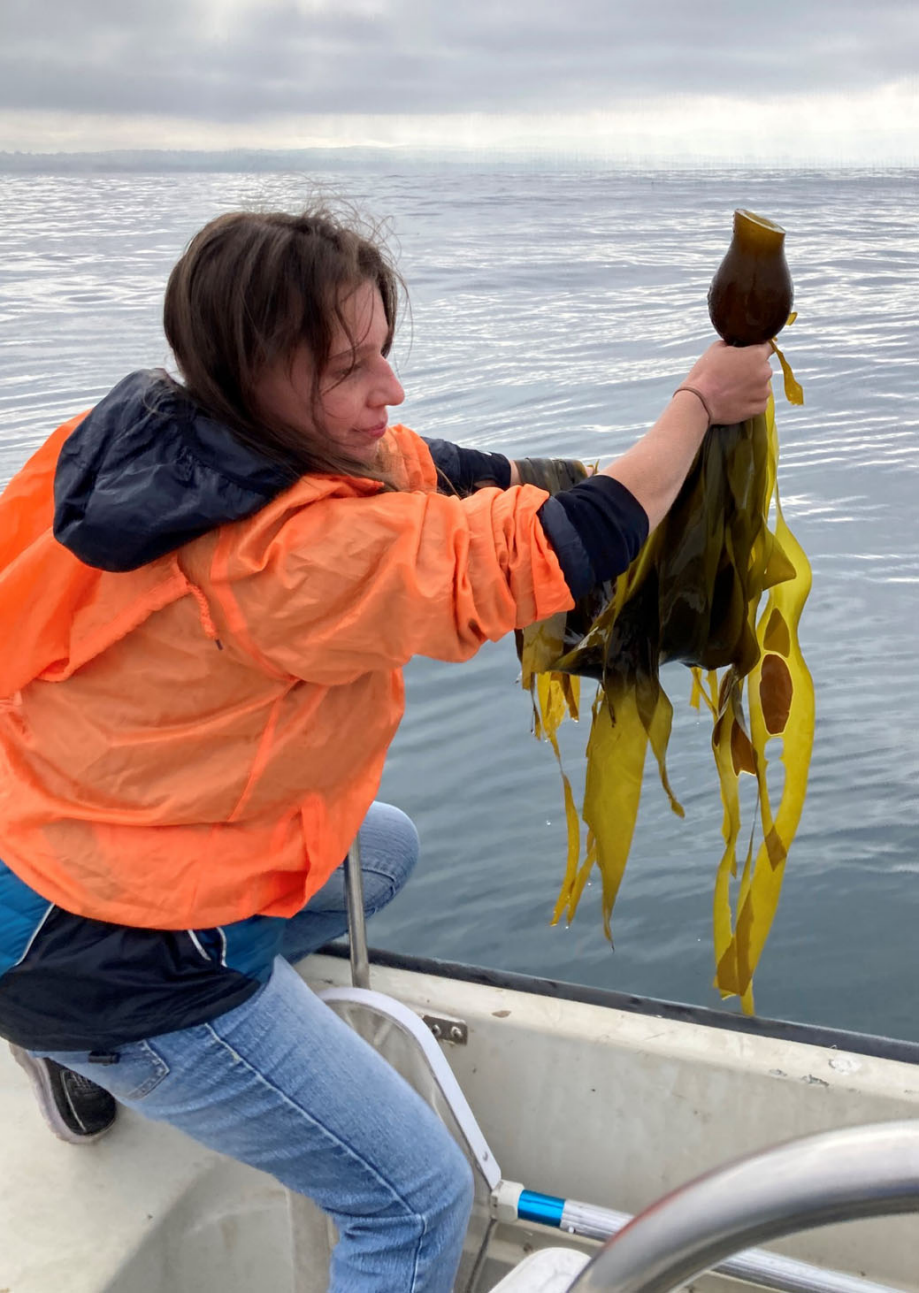


**Ekaterina Gribkova**

**Describe your scientific journey and your current research focus**

Throughout my scientific journey, I have always been driven by a passion for interdisciplinary work. During my undergraduate studies at the University of Illinois at Urbana-Champaign (UIUC), I pursued a double major in mathematics and molecular and cellular biology. My first undergraduate research experience was in a lab specialising in auditory neuroscience, which allowed me to develop skills in both computational modelling and neurophysiology, leading into the start of my PhD. During that time, I attended a summer course for methods in computational neuroscience at the Marine Biological Laboratory, which broadened my expertise and perspective, leading me to later join a systems neuroethology lab at UIUC. Today, as a postdoctoral researcher, my work spans neurobehavioral studies, computational modelling of memory, plasticity, and behaviour, and the development of biologically inspired artificial intelligence.

**Who or what inspired you to become a scientist?**

While I became interested in mathematics and biology very early on, inspired by my family, nature trips, and middle-school Science Olympiad, I think what ultimately cemented my resolve for becoming a scientist is the thrill of discovery. Our study of octopus hypnosis is a particularly exciting example, as we ventured into an area that had been largely forgotten for over a century.

**How would you explain the main finding of your paper?**

Octopuses are fascinating animals with arms that can act almost independently of the brain. To study how their arms work, scientists need the octopus to stay still, but common methods of general anaesthesia affect the whole nervous system, making it hard to study local arm behaviours. To address this, we revisited and improved a largely unknown technique of ‘octopus hypnosis’ (also known as octopus tonic immobility), which was first described more than a century ago by Vasily Danilewsky, and then J. Ten Cate. Essentially, this is a method of inducing a calm, sleep-like state in an octopus, by keeping it mouth-side up. Unlike general anaesthesia, this octopus hypnosis method preserves local arm functions, thus aiding in certain electrophysiological and behavioural studies that explore how octopus arms and suckers coordinate.

… our approach provides new opportunities for advancing both neurobehavioral research and octopus laboratory welfare.

**What are the potential implications of this finding for your field of research?**

Our modified method of octopus hypnosis/tonic immobility enables more precise studies of octopus arm behaviours and peripheral neural control, which may offer insights for comparative neurobiology and inspire distributed control algorithms in soft robotics and artificial intelligence. Our observation that octopus hypnosis is less effective in senescent female octopuses also raises some interesting questions about neurobehavioral mechanisms of tonic immobility and changes during octopus senescence. Thus, our approach provides new opportunities for advancing both neurobehavioral research and octopus laboratory welfare.

**Figure:**
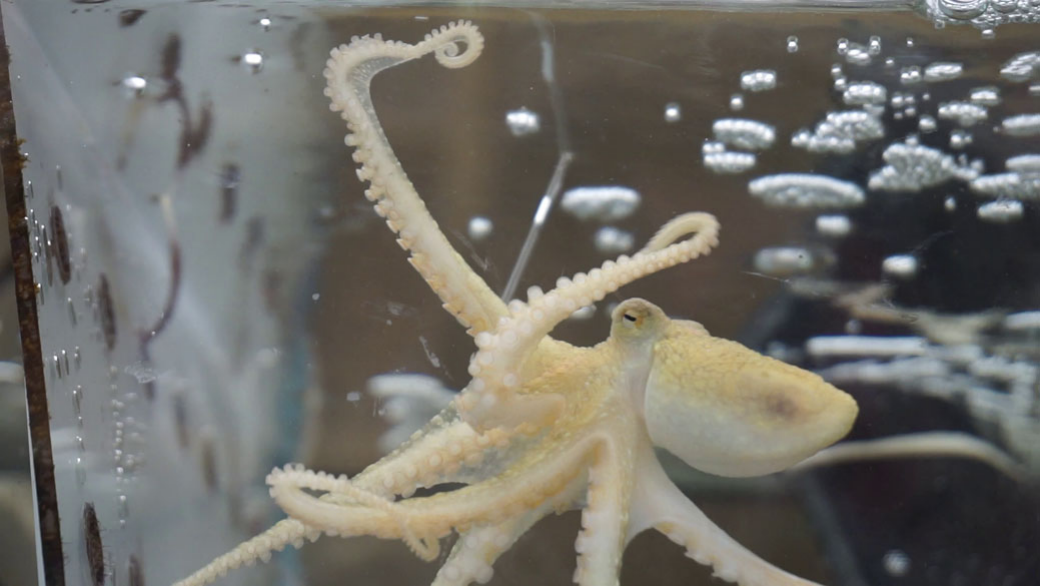
Photo of an octopus reacting to chemotactile stimuli.

**Which part of this research project was the most rewarding?**

Our first experiment was quite unforgettable. While conducting octopus arm sensorimotor studies and searching for older studies, we stumbled upon a blog post referencing ‘octopus hypnosis’ (ZPi | How To Hypnotize An Octopus), which was the first time we had ever heard of it. With very limited information about the method (as the original 1890 and 1928 studies were not readily available at the time) and some modifications, we turned our small *Octopus rubescens* mouth-side up in the water, and we were amazed to see that the octopus eventually started to stay still, almost as if it were falling asleep! That moment of surprise and discovery was incredibly rewarding and set the stage for the entire project.

**What do you enjoy most about being an early-career researcher?**

I enjoy the freedom of exploring new ideas and the thrill of interdisciplinary work. At this stage, I collaborate across many fields and combine my expertise in biology, neuroscience, and computational modelling in innovative ways. Every project feels like an exciting opportunity to push boundaries and learn something unexpected.

**What piece of advice would you give to the next generation of researchers?**

Particularly in context of our current paper, I would say: look back at older research and preserve it, even if it's not well-known. Don't let older ideas and methods get lost to time but also re-examine them with what we now know. Many of our discoveries would not have been possible without this.

**What's next for you?**

Ultimately, with my interdisciplinary research and teaching, I plan to continue bridging experimental biology with computational frameworks to understand and replicate adaptive intelligence.
